# NCAM1/FGF module serves as a putative pleuropulmonary blastoma therapeutic target

**DOI:** 10.1038/s41389-019-0156-9

**Published:** 2019-09-02

**Authors:** Rachel Shukrun, Hana Golan, Revital Caspi, Naomi Pode-Shakked, Oren Pleniceanu, Einav Vax, Dekel D. Bar-Lev, Sara Pri-Chen, Jasmine Jacob-Hirsch, Ginette Schiby, Orit Harari-Steinberg, Michal Mark-Danieli, Benjamin Dekel, Amos Toren

**Affiliations:** 1 Pediatric Stem Cell Research Institute, Safra Children’s Hospital, Sheba Medical Center, 5262000 Ramat-Gan, Israel; 2 Sackler School of Medicine, Tel Aviv University, 6997801 Tel Aviv, Israel; 3 Pediatric Hematology Oncology Research Laboratory, Safra Children’s Hospital, Sheba Medical Center, 5262000 Ramat-Gan, Israel; 4 Dr. Pinchas Borenstein Talpiot Medical Leadership Program 2013, Sheba Medical Center, Tel Hashomer, 5262000 Ramat-Gan, Israel; 5 The Maurice and Gabriela Goldschleger Eye Research Institute, Sheba Medical Center, 5262000 Ramat-Gan, Israel; 6 Cancer Research Center and the Wohl Institute of Translational Medicine, Sheba Medical Center, 5262000 Ramat-Gan, Israel; 7 Department of Pathology, Sheba Medical Center, 5262000 Ramat-Gan, Israel; 8 Division of Pediatric Nephrology, Safra Children’s Hospital, Sheba Medical Center, 5262000 Ramat-Gan, Israel

**Keywords:** Cancer models, Cancer stem cells, Paediatric cancer

## Abstract

Pleuropulmonary blastoma (PPB) is a rare pediatric lung neoplasm that recapitulates developmental pathways of early embryonic lungs. As lung development proceeds with highly regulated mesenchymal-epithelial interactions, a DICER1 mutation in PPB generates a faulty lung differentiation program with resultant biphasic tumors composed of a primitive epithelial and mesenchymal stroma with early progenitor blastomatous cells. Deciphering of PPB progression has been hampered by the difficulty of culturing PPB cells, and specifically progenitor blastomatous cells. Here, we show that in contrast with in-vitro culture, establishment of PPB patient-derived xenograft (PDX) in NOD-SCID mice selects for highly proliferating progenitor blastoma overexpressing critical regulators of lung development and multiple imprinted genes. These stem-like tumors were sequentially interrogated by gene profiling to show a FGF module that is activated alongside Neural cell adhesion molecule 1 (NCAM1). Targeting the progenitor blastoma and these transitions with an anti-NCAM1 immunoconjugate (Lorvotuzumab mertansine) inhibited tumor growth and progression providing new paradigms for PPB therapeutics. Altogether, our novel in-vivo PPB xenograft model allowed us to enrich for highly proliferating stem-like cells and to identify FGFR and NCAM1 as two key players that can serve as therapeutic targets in this poorly understood and aggressive disease.

## Introduction

Pleuropulmonary blastoma (PPB) is the most common primary malignancy of the lungs in children^1^. It is a rare and highly aggressive tumor of the Pleuropulmonary mesenchyme that arises during fetal lung development and occurs most often in infants and children younger than 10 years^1,2^. Despite a multimodality treatment approach, outcomes are uniformly poor with an overall 2-year survival rate of 63%^3^. Therefore, there is an urgent need to uncover novel therapeutic strategies. This embryonal tumor of the lung is characterized by a multi-step tumor progression from a less aggressive to a more aggressive phase via a sequence of morphological changes, reflecting biological progression and predicting clinical outcome^4^. The early stage of PPB (Type I PPB) is characterized by the presence of epithelial cysts and small numbers of uncommitted mesenchymal cells. In later stages of tumorigenesis, the mesenchymal cells expand and overgrow the epithelial cysts, forming an overtly malignant cystic and solid (Type II) or purely solid sarcoma (Type III)^5^. The malignant cellular component of PPB is a high-grade sarcoma that derives from an immature lung mesenchymal cell that has the capacity to differentiate into multiple mesenchymal lineages. It has been shown that in approximately 70% of cases, PPB appears to develop as the result of inherited germline mutations in the microRNA-processing enzyme DICER1^5–8^.

The use of patient derived xenograft (PDX) model systems for studying cancer has gained great popularity in recent years. These in-vivo models provide unique opportunities to uncover and explain important cancer-related cellular pathways, and have therefore become the reference model for functional validation of discoveries in the field of tumor biology and for preclinical evaluation of anticancer therapy. The robustness and reproducibility of the assay, together with the remarkable preservation of the characteristics of the tumor of origin, make PDX a trustable surrogate of patient tumor for many types of cancers. In our lab we have used a PDX model to study cancer stem cells (CSCs) populations and define processes involved in tumor initiation and progression of several rare pediatric tumors, including Wilms’ tumor (WT)^9–11^, Angiomyolipma^12^ and malignant rhabdoid tumors^13^. We have shown that serial PDX propagation in mice significantly enriches for CSC function and results in a more aggressive phenotype in late passage xenograft (Xn), thereby unveiling responsible pathways and molecules that can serve as new therapeutic targets^10,11,14–17^.

Herein, we have established a PPB PDX model, which simulates the natural history of PPB progression, thereby providing a unique platform to uncover and explain biological processes that occur during disease progression. We used this model to dissect tumor biology and uncovered a novel therapeutic target for PPB, namely Neural cell adhesion molecule 1 (NCAM1). In addition, this model can serve as a renewable laboratory resource for discovering and testing potential therapeutic targets in this rare pediatric malignancy.

## Results

### PPB serial propagation is associated with increased tumor aggressiveness

**Fig. 1:**
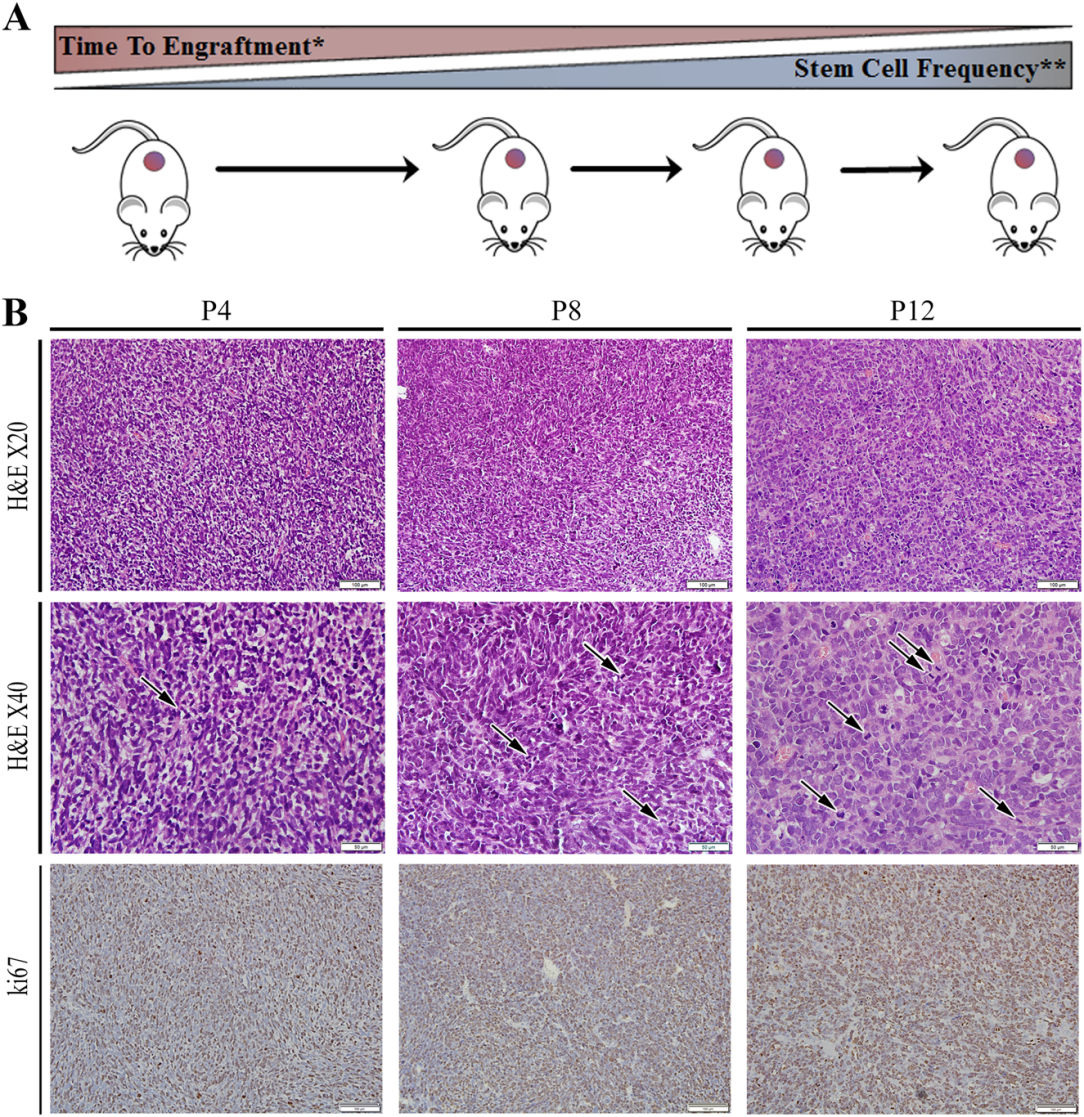
Long-term propagation of PPB is associated with increased tumor aggressiveness. **a** Generation of PPB PDX model (Scheme): serial propagation of human PPB Xn in NOD/SCID mice resulted in shorter time to tumor engraftment and progression and enrichment of CSC population along serial passages. *p = 0.02 **p = 0.009. See also Tables 1 and 2. **b** PPB H&E tissue staining along passages demonstrating morphological differences in late passage PDX including the acquisition of hypercellular primitive blastemal like cell morphology with multiple mitoses (black arrows indicate mitoses) with loss of cystic, and spindle cell appearance (top panels). KI67 IHC staining demonstrating increased proliferation capacity in high tumor passages (30–40% in P4, 40–50% in P8 and 55–60% in P12). Quantification of Ki-67 staining was performed by calculating the percentage of positive cells in five high power fields from different areas of the tumor (bottom panel); Scale bar; 100 μm (top and bottom panels), 50 μm (middle panel)

A human Pleuropulmonary blastoma sample was transplanted into NOD/SCID mice, thereby generating a PDX. Sequential propagation of PPB Xn in mice was performed by 1 × 10^6^ PDX derived cells injections. Serial propagation allowed us to establish early (P1–P7) and late-passage (P7-P14) PPB PDX that were studied for pathogenic pathways associated with PPB-initiating capacity. Sequential propagation of PPB PDX correlated with significantly shorter time to tumor engraftment and accelerated tumor growth (Table 1 and Fig. 1a), indicating the promotion of tumor aggressiveness along passages. We next queried whether CSC capacity is functionally enhanced with PPB propagation. We performed limiting dilution xenotransplantation experiments with PPB cells derived from early-passage and late-passage PDX. This analysis showed significant positive selection for CSC frequency in late-passage PDX (Table 2 and Fig. 1a), as evident by the significantly increased engraftment frequency. Having observed that high-passage PPB PDX selects for the CSC population, we analyzed histological and immunohistochemical changes that accompany the acquisition of the CSC phenotype along passages. H&E staining revealed that PDX derived tumors maintain the basic mesenchymal PPB cellular morphology. Nevertheless, some morphological differences were observed in late passage PDX including the acquisition of a hypercellular primitive blastemal like cell morphology with multiple mitoses while losing cystic and spindle cell appearance. (Fig. 1b). In addition, we carried out KI67 immunohistochemical (IHC) staining, demonstrating increased proliferation in high-passage tumors (Fig. 1b). Mouse cell tumor contamination was ruled out by HLA IHC staining and FACS analysis of the mice specific antigen H2K (Fig. S1A and S1B accordingly). In addition, STR analysis demonstrated identical genetic signature comparing several samples including primary tumor, P2, P8, and P12 (Fig. S1C).

**Table 1:** PPB PDX frequency and characteristics during propagation

	Engraftment rate (%)	Time to engraftment (Days)*	Time to resection (Days)	Average weight	Average volume
Early	63.16	58.08	77.57	1.43	0.88
Late	87.50	33.85	51.94	1.83	1.03

Table summarizing the frequency and characteristics of secondary tumor formation from PPB PDX and further propagation. During serial propagation of PPB PDX shorter time to tumor engraftment and accelerated tumor growth were noticed. In the comparison between early and late PDX passages time to engraftment

*p = 0.02, Mann–Whitney *U* test

**Table 2:** Limiting dilution xenotransplantation summary representing tumor CSC frequency during propagation

	Number of cells injected	Engraftment rate	Stem cell frequency*
Early	1000	3/4	**1/1581**
	500	0/4	
	100	0/4	
Late	1000	3/4	**1/304**
	500	4/4	
	100	2/4	
	50	/14	
	10	0/4	

Summary of CSC frequency estimated by limiting dilution xenotransplantation of cells isolated from early and late PDX passages. The data is presented as the ratio of injections that formed tumors within 12 weeks. Bold numbers represent the estimated CSC frequency. In the comparison between early and late PDX passages. Estimation of the relative frequency of cancer propagating cells was calculated online using the ELDA software online (http://bioinf.wehi.edu.au/software/elda/)

*p = 0.009

### Sequential PDX propagation model generated putative biomarker molecules involved in tumor aggressiveness

**Fig. 2:**
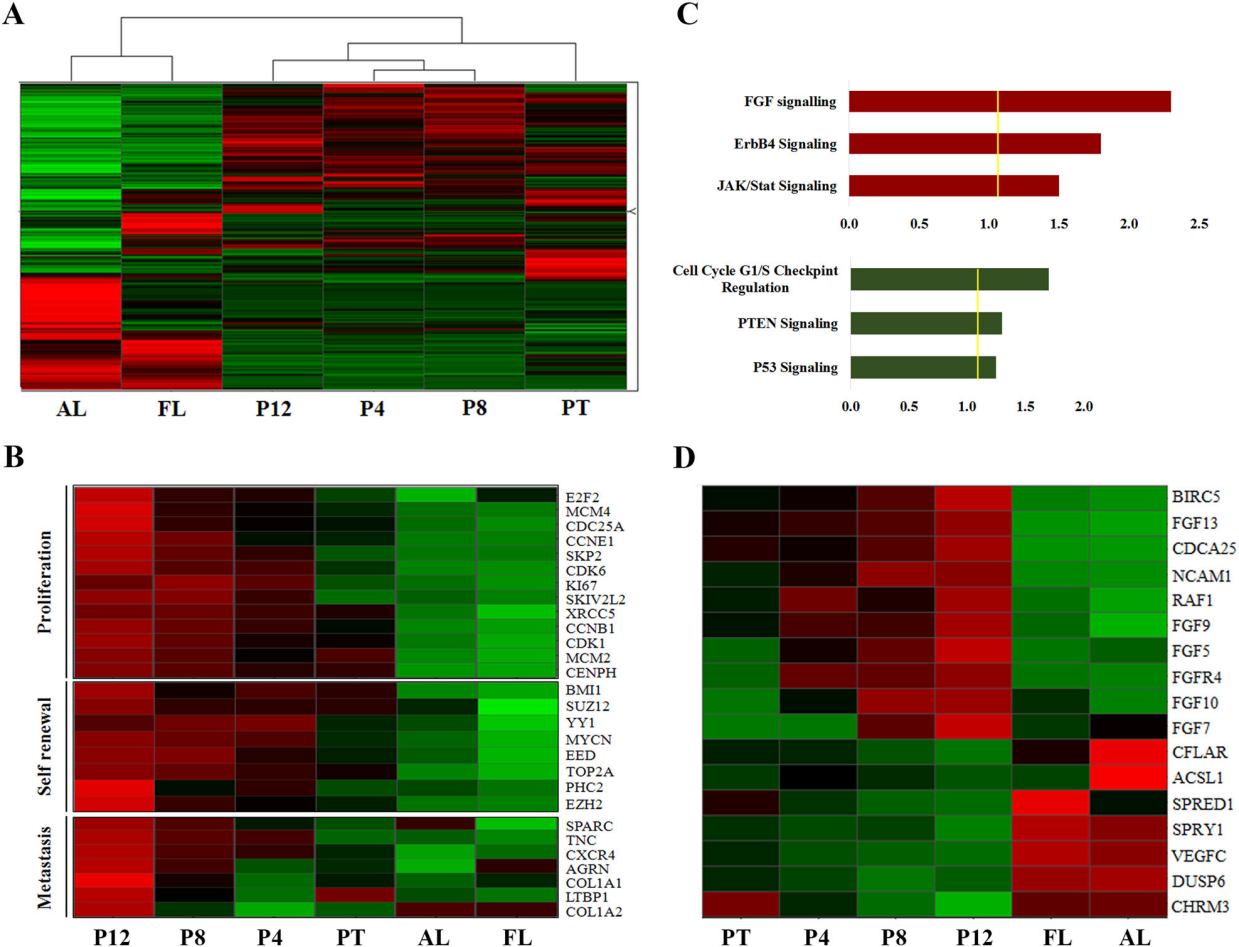
Global gene signature reveals putative biomarkers involved in tumor aggressiveness. **a** Microarray gene expression analysis comparing the different samples: 1. Primary PPB (PT); 2. Early PPB PDX (Passage 4-P4); 3. Intermediate PPB PDX (Passage 8-P8); 4. Late PPB PDX (Passage 12-P12); 5. Adult lung (AL); 6. Fetal lung (FL), reveals resemblance between PT and its derived Xn samples in comparison to adult and fetal normal lung tissues. **b** Gene heat map reveals high expression of proliferation genes in later passages (e.g., *KI-67, CDK1*, and *E2F2*), upregulation of self-renewal genes (e.g., *BMI1, TOP2A*, and *EZH2*), and an invasive gene signature (e.g., *SPARC, CXCR4*, and *TNC*) in late passage PDX. **c** Ingenuity® comprehensive pathway and network analysis reveals that FGF signaling pathway is one of the most upregulated pathway in late aggressive PDX passages, among the most downregulated pathways are cell cycle checkpoints regulation, PTEN and P53 pathways. **d** Gene heat map revealed upregulation of genes that activate the FGF signaling pathway and FGF pathway target genes (e.g., *FGF5, FGF7, FGF10, BIRC5*, and *RAF1*), as well as a low expression of genes that downregulate the FGF signaling pathway (e.g., *CFLAR, SPRY1, ACSL1*, and *DUSP6*) in late passage PDX in comparison to early passages PDX

We next sought to characterize the global molecular profile of sequential PPB PDX. For this purpose, we performed a microarray gene expression analysis comparing several different samples: (1) primary PPB (PT); (2) Passage 4 (P4); (3) Passage 8 (P8); (4) Passage 12 (P12); (5) Adult lung (AL); (6) Fetal lung (FL). Unsupervised hierarchical clustering revealed a resemblance between PT and it’s derived Xn samples in comparison to adult and fetal normal healthy lung tissues (Fig. 2a). Gene expression analysis of the samples demonstrated an early mesenchymal developmental signature in late Xn passages (P12) including the upregulation of critical regulators of lung formation (e.g., *GLI2, GLI3, PITX2, LEFTY, SOX11, SOX8*) alongside paternally expressed genes (e.g., *PEG1/MEST, PEG3, PEG10, NNAT, KCNQ1OT1, DLK1*, and *IGF2)* as previously shown for the WT blastemal^14^ (Table S1). In addition, Ingenuity® functional analysis comparing late passage Xn vs. normal adult lung, demonstrated that among the most upregulated pathways in P12 are several developmental pathways including embryonic and respiratory development (Fig. S2A).

Moreover, gene heat map analysis revealed high expression of proliferation-related genes in late passages (e.g., *KI67, CDK1*, and *E2F2*), as well as upregulation of self-renewal genes (e.g., *BMI1, TOP2A*, and *EZH2*), with an invasive gene signature (e.g., *SPARC, CXCR4*, and *TNC*), predicting metastatic behavior (Fig. 2b). Thus, the sequential PDX propagation model selected highly aggressive tumor traits, generating a platform for putative biomarker molecules involved in tumor progression/aggressiveness.

### NCAM1 and FGF are PPB key regulatory molecules

To identify key regulatory pathways active in PPB samples, we next preformed Ingenuity® comprehensive pathway and network analysis comparing the different PDX passages. The analysis revealed that the FGF signaling pathway is one of the most upregulated pathways and that the cell cycle checkpoints regulation PTEN and P53 pathways are among the most downregulated pathways in late aggressive Xn passages (Fig. 2c). Close look at the microarray data showed that among the most upregulated genes in late passage PDX were genes that activate the FGF signaling pathway (e.g., *FGF5, FGF7*, and *FGF10*). Moreover, activation of several FGF downstream signaling pathways was also observed including cancer related pathways (RAS-MAPK, PIK3-AKT, and STAT) with a significant upregulation of several key molecules in these pathways (e.g., *BIRC5, RAF1, MYCN, IGF2, SNAI2, POSTN*) (Fig. 2d and Table S2). Finally, a low expression of genes downregulating the FGF signaling pathway was demonstrated (e.g., *CFLAR, SPRY1, ACSL1*, and *DUSP6*) (Fig. 2d).

Having observed that our molecular screen independently pinpointed the FGF signaling pathway, we looked for related membrane expressed molecule that could serve as a putative therapeutic target. As previously demostrated, FGFRs can also be activated by non FGF ligands such as NCAM1. The functional interaction between NCAM1 and FGF signaling pathway was originally reported in neurons^18^. Thereafter, extensive evidence of a physical association between the two proteins in different, non‐neural cell types, was demonstrated^19–22^. Accordingly, Ingenuity analysis of our microarray data demonstrated an interaction between NCAM1, FGF receptors and several of their downstream targets (Fig. S2B). Importantly, NCAM1 has been suggested as a CSC marker and a therapeutic target in other pediatric solid tumors^11^, and could therefore validate our approach.

**Fig. 3:**
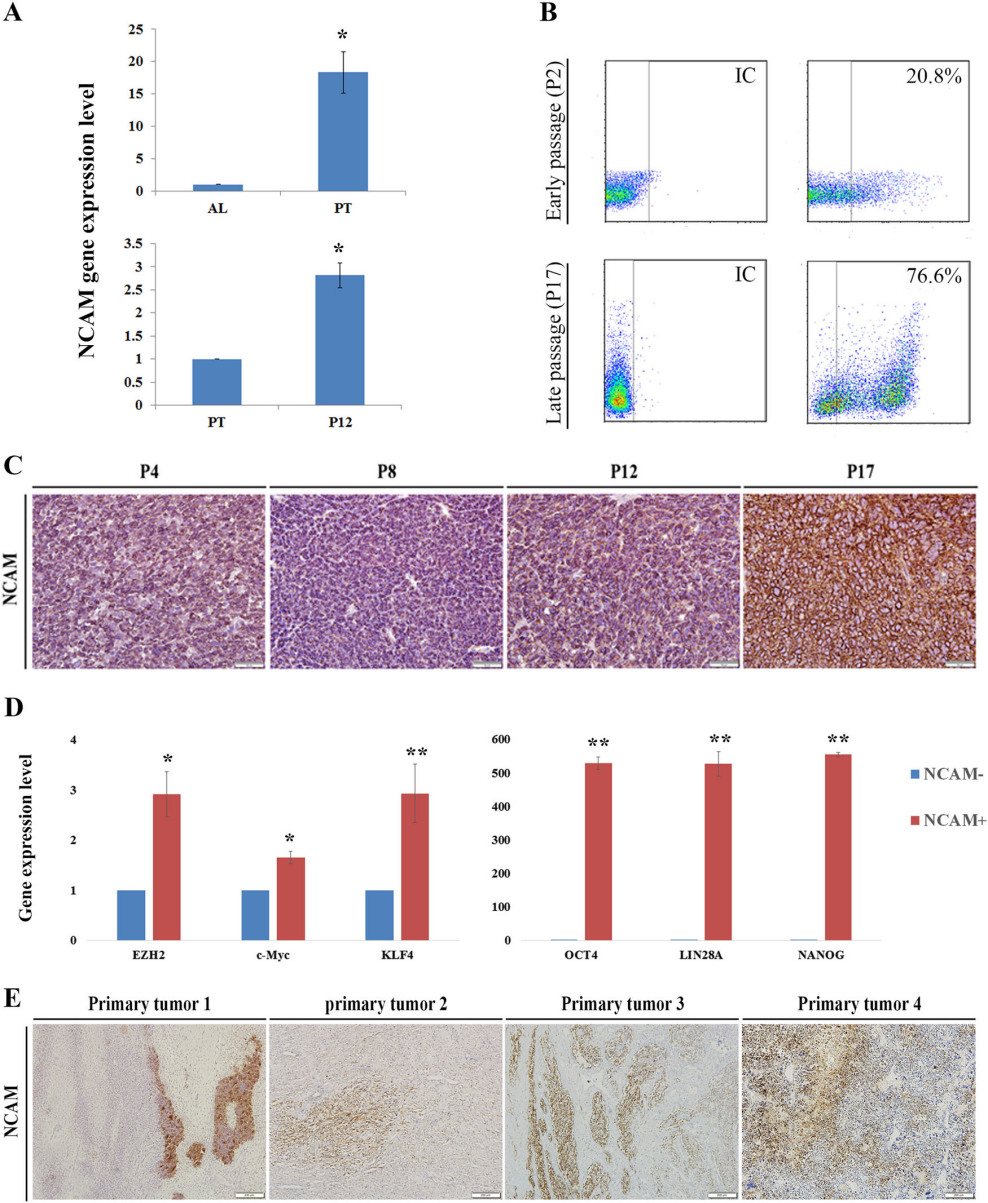
NCAM1 is a PPB key regulatory molecule. **a** qRT-PCR analysis revealed high NCAM1 expression in primary PPB tumor (*n* = 3) in comparison to healthy adult lung control sample (*n* = 3) (top) and in late passages. (*n* = 3) in comparison to primary tumor (bottom); *p < 0.01; Mann–Whitney *U* test. **b** FACS analysis revealed that in early passage PDX cells (P2), only 20% of cells were NCAM1^+^ in comparison to 76% of cells in late passage (P10). **c** IHC staining demonstrating an increased expression of NCAM1 along the passages; Scale bar, 100 μm. **d** qRT-PCR analysis reveals high gene expression of several self-renewal genes (e.g., *BMI1, EZH2*, *OCT4* and *KLF4*) in the NCAM1^+^ cells; For qRT-PCR analyses the values of primary tumor cells were used to normalize (therefore = 1) and all other values were calculated accordingly. Results are presented as the mean ± S.E.M of three separated experiments. *p < 0.05, **p < 0.01. **e** IHC staining demonstrating NCAM1 expression in four different PPB primary tumors; Positive NCAM1 expression was estimated at 10% (tumors 1 and 2), 20% (tumor 3), and 30% (tumor 4) of primary tumor cells. Scale bar, 100 μm

Analysis of the microarray data showed an increased NCAM1 expression along the passages (Fig. S3). In order to validate our results, we next preformed qRT-PCR analysis that revealed high NCAM1 expression in primary PPB tumor compared to healthy adult lung control samples (top) and in late passages PPB-Xn in comparison to primary PPB (bottom) (Fig. 3a). Next, we carried out FACS analysis to quantify the proportion of the NCAM1^+^ population in the different stages of PDX propagation. This analysis revealed that in early passage PDX cells (P2), only 20% of cells were NCAM1^+^ in comparison to 76% of cells in late passage (P10) (Fig. 3b). Finally, immunohistochemistry staining demonstrated increased expression of NCAM1 and several FGF signaling molecules (i.e., FGF5 and FGF7) along passages, emphasizing the relevance of our approach (Fig. 3c and Fig. S4 accordingly).

We next sorted NCAM1 positive cells in order to examine gene expression patterns in NCAM1^+^ vs. NCAM1^−^ sorted cells. qRT-PCR analysis revealed high expression of several self-renewal genes (e.g., *BMI1, EZH2*, *OCT4*, and *KLF4*) in the NCAM1^+^ population, supporting their stem cell phenotype (Fig. 3d). We next sought to examine whether NCAM1 and FGF key signaling molecules are expressed in other primary PPB tumors. Indeed, IHC staining demonstrated positive NCAM1 and FGF5, FGF7, and FGF10 expression in several PPB primary tumors, validating the relevance of our findings (Fig. 3e and Fig. S5 accordingly).

### NCAM1 as a PPB therapeutic target

Based on our observation that NCAM1 expression increased during Xn propagation, we next sought to establish NCAM1 as a therapeutic target in an in-vitro model. To do so, we examined the therapeutic effects of lorvotuzumab mertansine (huN901-DM1), a humanized anti-NCAM1 antibody-cytotoxic drug conjugate, on PPB PDX cell morphology and gene expression. huN901-DM1 is an antibody-drug conjugate (ADC), consisting of a humanized anti-CD56 antibody to which the tubulin-binding maytansinoid DM1 is covalently conjugated via a stable disulfide linker^23^. huN901-DM1 targets CD56 at the cell surface and, upon antigen binding, becomes internalized, resulting in the intracellular release of DM1^24^, which in turn promotes disruption of microtubule assembly, G2/metaphase arrest, and ultimately apoptosis^25,26^.

**Fig. 4:**
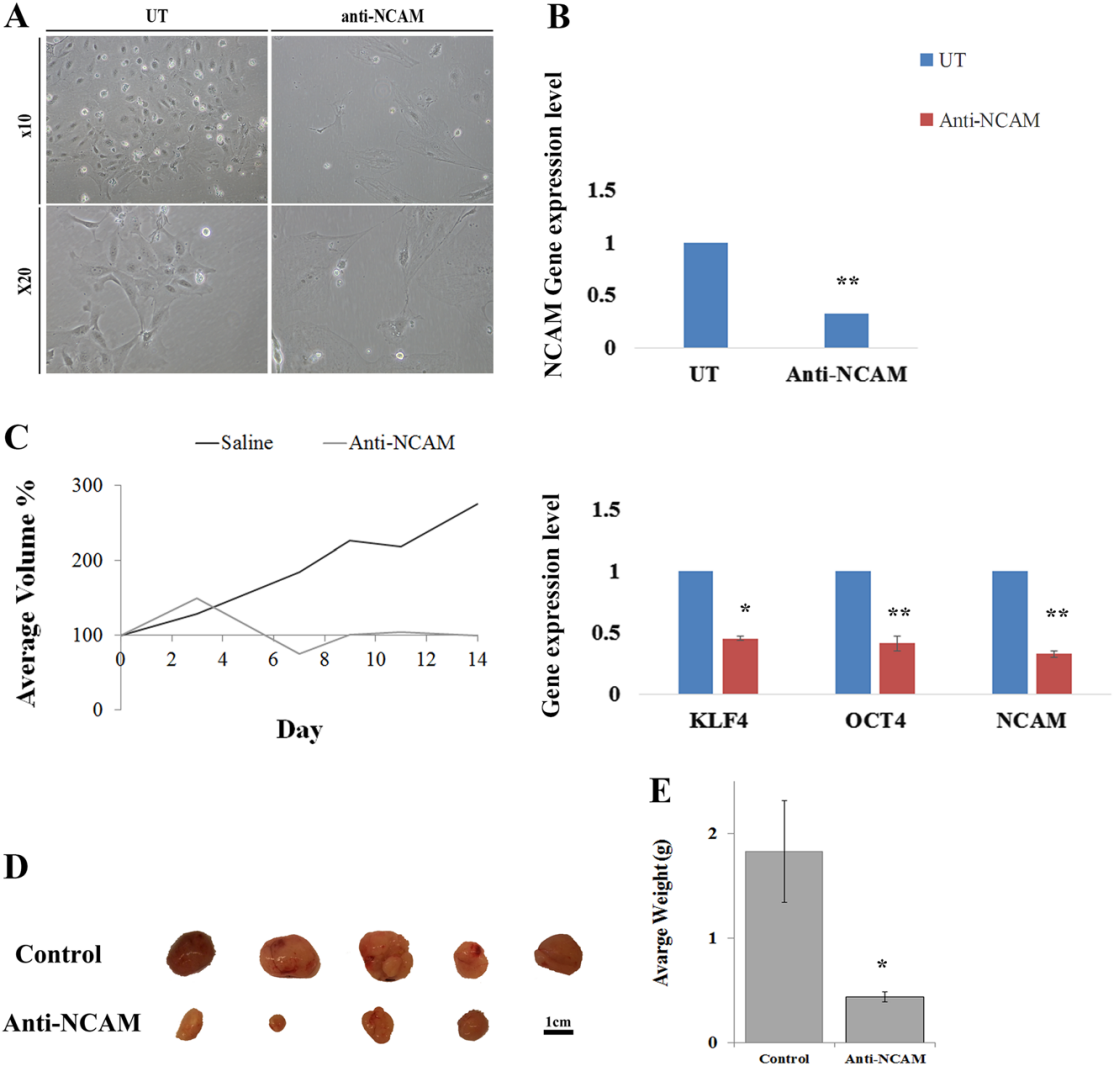
Functional validation of NCAM1 as a possible PPB therapeutic target. **a** Following 5 days of treatment with a humanized anti-NCAM1 antibody-cytotoxic drug conjugate on PPB PDX cells, we observed changes in cell morphology. Anti-NCAM1 treated cells were found to be larger, fewer in number and with more cell fragments compared to untreated cells. **b** qRT-PCR analysis on treated (*n* = 3) and untreated (*n* = 3) tumor cells revealed a significant downregulating of NCAM1 expression (top), as well as downregulation of several self-renewal genes (e.g., *LIN28A, OCT4*, and *KLF4*) (bottom) in the anti-NCAM1 treated cells; For qRT-PCR analyses the values for un-treated cells were used to normalize (therefore = 1) and all other values were calculated accordingly. Results are presented as the mean ± S.E.M of three separate experiments. *p < 0.01. **c**, **d** PPB PDX were formed and randomly divided into two groups, first group (*N* = 4) was treated with huN901-DM1 with a dosage of 360 µg/Kg, while the second group (*N* = 5) was treated with saline as a control. Mice were treated intravenously twice weekly for a fortnight on days 0, 3, 7, and 11, and were observed for 17 days. In the Anti-NCAM1 treatment group both external tumor volume measured during treatment (**c**) and tumor volume measured following animal sacrificing (**d**) were significantly lower in comparison to tumor volume in the control group; *p* < 0.05, Mann–Whitney *U* test. **e** Tumor weight measurements following tumor removal demonstrated significantly lower weights in the Anti-NCAM1 treated group compared to the control group; *p < 0.05, Mann–Whitney *U* test

**Fig. 5:**
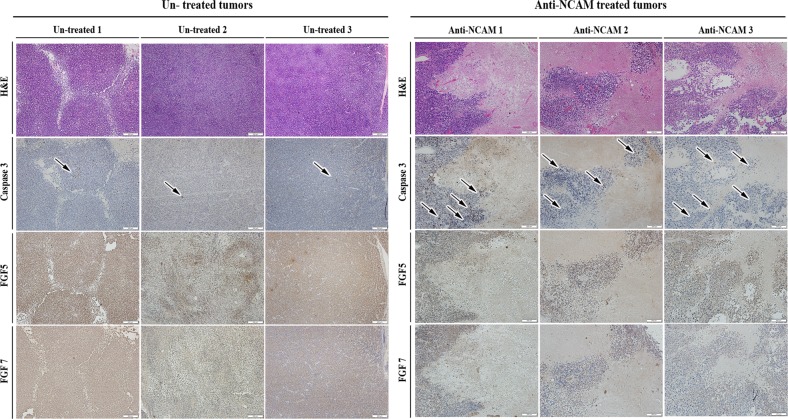
IHC characterization of un-treated PPB tumor samples (left panel) vs. PPB tumor samples treated with anti-NCAM antibody (right panel). PPB H&E tissue staining demonstrated vast areas of necrosis following treatment (top panel); Immunohistochemical staining of cleaved caspase-3 demonstrating increased staining in treated tumor samples (black arrows indicate high caspase-3 expressing cells), (middle panel); FGF5 and FGF7 IHC staining showed downregulation of these key FGF pathway molecules in anti-NCAM treated tumor samples (bottom 2 panels); Scale bar; 200 μm

First, cells were treated in-vitro with anti-NCAM1 antibody-cytotoxic drug conjugate for 5 days. Following the treatment, cell morphology was examined and compared to untreated cells (Fig. 4a). Anti-NCAM1 treatment resulted in increased cell death and apoptotic cell features, including cell swelling and fragmentation. Next, we performed a qRT-PCR analysis on treated and untreated tumor cells revealing significant downregulating of NCAM1 expression, as well as downregulation of several self-renewal genes (e.g., *LIN28A, OCT4*, and *KLF4*) in the anti-NCAM1 treated cells (Fig. 4b). We then turned to examine the therapeutic effects of anti-NCAM1 antibody-cytotoxic drug conjugate on PPB PDX progression capacity in-vivo. For this purpose, nine PPB Xn were formed and randomly divided into two groups, 4 were treated with huN901-DM1, while 5 were treated with saline as a control. We observed a significant difference in tumor growth rate in anti-NCAM1 antibody treated mice vs. the control group following treatment (Fig. 4c–e and Fig. S6A). A minor decrease in weight was observed in the huN901-DM1 treated group, while an eight percent weight gain was observed in the control group, most likely attributed to the significant increase in tumor volume. (Fig. S6B). There was no effect on viability nor on morbidity. To examine whether huN901-DM1 targeted preferentially NCAM1 expressing cells within the cell population, we compared NCAM1 expression in the treated and control cell population using FACS analysis. We observed a significant downregulation in NCAM1 expression after treatment in the treated cells compared to the control group, 1.65% vs. 95.36% NCAM1, respectively (Fig. S6C). Finally, we performed IHC characterization of un-treated PPB tumor samples in comparison to PPB tumor samples following anti-NCAM treatment (Fig. 5). PPB H&E tissue staining demonstrated vast areas of necrosis following treatment. In addition, IHC staining of cleaved caspase-3 showed increased staining in treated tumor samples, indicating a significant apoptotic activity following treatment. Next, we performed FGF5 and FGF7 IHC staining that revealed downregulation of these key FGF pathway molecules in anti-NCAM traeted tumor samples (Fig. 5). This data strengthens our earlier findings, implicating NCAM1 as a therapeutic target.

## Discussion

PPB represents a highly aggressive yet poorly understood malignancy, which to date has been extremely difficult to study, as there are no relevant cell lines or animal models at hand. In this paper, we describe the establishment of a novel PPB-PDX model that is expected to serve as a renewable tissue resource and provide a clinical model for studying and targeting this rare but lethal pediatric neoplasm. Altogether, our results show that serial in-vivo passaging of PPB PDX selects for a highly proliferative population, strongly correlates with an aggressive phenotype and is accompanied by upregulation of several proliferation and self-renewal genes. Moreover, among the most upregulated genes were NCAM1 and its related partners, the FGFRs, belonging to the FGF signaling pathway.

In the present study we suggest a possible role for NCAM1 and the FGF signaling pathway in PPB progression. Our data revealed increased expression of both NCAM1 and FGFs in tumor samples in comparison to normal adult lung and along PDX propagation. Upregulation of several FGFRs (FGFR1, FGFR2, and FGFR4) and their ligands FGF5, FGF7, FGF9, FGF10, and FGF13 was also demonstrated along with activation of major pathways downstream of activated FGFRs including RAS-MAPK, PIK3-AKT, and STAT.

FGF and its four FGFRs (FGFR1-4) regulate a multitude of cellular processes including cell growth, differentiation, migration and survival, and have been implicated in a number of physiological and pathological processes including angiogenesis, wound healing and cancer^27,28^ Interestingly, FGFRs can also be activated by non FGF ligands such as the cell adhesion molecules L1, NCAM1^29^, which was also overexpressed in our PDX PPB model. NCAM1 is considered a signaling receptor that impacts cellular adhesion, migration, proliferation, apoptosis, differentiation, survival, and synaptic plasticity^30^. In human diseases, NCAM1 has been expressed in various tumors of the nervous system, malignant NK/T-cell lymphomas and neuroendocrine carcinoma^31–33^. The activities of NCAM1 are mediated both by homophilic (NCAM to NCAM) and heterophilic binding to other adhesion molecules, extracellular matrix components and cell surface receptors^34^. Among the heterophilic partners of NCAM1, FGFR has attracted the attention of many investigators due to its functional implications.

The functional interaction between NCAM1 and FGFR was originally reported in neurons^18^. Thereafter, extensive evidence of a physical association between the two proteins in different, non‐neural cell types was demonstrated^19–22^. This data was further confirmed by studies in which direct binding of NCAM1 to FGFR1 and FGFR2 was revealed^35–37^. Based on the ability of NCAM to modulate FGFR function and on the proposed role of FGFR activity in cancer development, we hypothesized that the NCAM/FGFR signaling axis may have a role in PPB development, as in fact has been already shown in other tumors^38–42^. By demonstrating increased expression of NCAM1 and FGF/FGFRs in primary PPB in comparison to normal adult lung tissue and by demonstrating high NCAM1 expression in other primary PPB tumor biopsies, we reinforce our hypothesis that interplay between NCAM1 and FGFRs can contribute to PPB development and progression.

Interestingly, Dicer-null lung tissues showed increased expression of FGF10 protein with an expanded distribution in lung mesenchyme, further supporting our suggestion that upregulation of FGF signaling pathways may have a role in PPB tumorigenesis and progression. Of relevance, Harris and colleagues speculated that lack of miRNA control might account for the dysregulation of FGF10^43^. Moreover, it has been shown that FGF9 is overexpressed in lung epithelium in the initial multicystic stage of Type I PPB and that in mice lacking epithelial Dicer1, increased FGF9 expression results in pulmonary mesenchymal hyperplasia and a multicystic architecture that is histologically and molecularly indistinguishable from Type I PPB^44^.

To date, there is not a single basic science paper describing in vivo models of PPB allowing for a better understanding of this tumor, mostly because of how rare the tumor is, lack of available cell lines and difficulties culturing PPB cells. Using the serial propagation of PDX model, we were able to demonstrate the enrichment of CSC frequency and increased tumorigenicity through PDX passages. Our in-vivo model was previously used to propagate other aggressive and pediatric rare tumors^10–13^. The main novelty of our current study lies in the contribution of our in-vivo model to unravel the NCAM1 molecule and its suggested interplay with FGFR in the progression of PPB, pinpointing these molecules as possible novel therapeutic targets in PPB.

Indeed, targeting PPB PDX with the anti-NCAM1 antibody-cytotoxic drug conjugate huN901-DM1 resulted in a reduction in PPB PDX propagation rate and difference in gene signature between treated and untreated cells. Since the antitumorigenic effect of huN901-DM1 has been attributed to its cytotoxic conugate DM1, a member of the maytansinoids mitotic inhibitors^23^, our results probably reflect the killing of NCAM1-high cells by the cytotoxic component rather than direct effect of anti CD56 on downregulation of self-renewal genes including FGF pathway. Nevertheless, due to the known role of NCAM in regulating the FGF pathway through its association with FGFR that results in FGFR recycling at the cell surface^45,46^, sustained signaling^36^, and cancer progression^47^, we believe that downregulation of NCAM1 by the antibody interferes with the NCAM/FGFR interplay which may result in reduction of tumor aggressiveness and metastatic properties as was shown in other cancers^47^. Finally, our work highlights the translational relevance of this model in identifying targetable genes in general and of the anti-NCAM1 strategy in particular, implicating NCAM1 as a therapeutic target. Moreover, from the translational perspective, huN901-DM1 is currently being evaluated as monotherapy in children with various NCAM1-expressing solid tumors including: Wilms tumor, rhabdomyosarcoma, neuroblastoma, malignant peripheral nerve sheath tumor and synovial sarcoma. Among patients eligible for this study are also children with relapsed or refractory PPB (http://clinicaltrials.gov/show/NCT02452554).

Our results point to a possible NCAM/FGFR interplay as a novel mechanism underlying PPB malignancy that may represent a valuable therapeutic target. We intend to further explore this axis and its role in PPB. Moreover, there are several available FGFR inhibitors that are currently in early phases of clinical development^48^, and integration of these drugs with the anti-NCAM antibodies can serve as a novel and promising therapeutic approach. Furthermore, to validate our findings, we will establish additional PPB PDX, and we will use our in-vivo model to unravel other regulatory molecules and pathways that may have a role in PPB development and progression in order to find new therapeutic targets.

In summary, we have successfully generated a new in-vivo model for PPB, a rare and fatal childhood malignancy, and have shown that this model can be used effectively to pinpoint key molecular pathways driving tumorigenesis and propagation. In turn, these can be harnessed to formulate targeted therapies for better tumor eradication. Specifically, we identified FGFR and NCAM1 as two key players, which could have significant implications on the effectiveness of treatment in this poorly understood disease.

## Materials and methods

### Ethics statement

This study was conducted according to the principles expressed in the Declaration of Helsinki and was approved by the Institutional Review Board of the Sheba Medical Center.

### Primary PPB and healthy pulmonary samples

Primary PPB sample was obtained from a lung tumor mass of a pediatric patient within 1 h of surgery. Informed consent was given by the legal guardians of the patient involved according to the declaration of Helsinki.

Fetal lung tissues for microarray expression analysis were taken from healthy aborted fetuses in 22 week gestational age and were kindly provided to us by Dr Chava Rosen (Weizmann Institute). Total RNA from normal human adult lung tissue was purchased from BioChain®.

### In vivo xenograft formation

The animal experiments were performed in accordance with the Guidelines for Animal Experiments of Sheba Medical Center. Initial PPB engrafting to 5–8 weeks old, female, nonobese diabetic immunodeficient (NOD/SCID) mice was performed as previously described^14^. Briefly, primary PPB tissue was cut into 2–5 mm pieces and implanted subcutaneously in the flank of the mouse. Tumors were harvested approximately 1–3 months post implantation or when they reached a size of 1.5 cm diameter. Time to engraftment, time to resection, weight and volume for each engrafted Xn were recorded. Xn tissue was immediately cut into small pieces and processed for further experiments as follows: (i) flash freezing for subsequent molecular characterization of extracted analyses; (ii) formalin fixation and paraffin embedding for future IHC studies. In addition, Xn serial passages were formed in two methods: (1) PPB Xn tissue was cut into 2–5 mm pieces and implanted subcutaneously in the flank of the mouse; (2) For the in-vivo experiments compering anti-NCAM1 antibody-cytotoxic drug conjugate vs. control and for the estimation of relative frequency of tumor propagating cells we injected dissociated cells from freshly retrieved PPB Xn; Cells were injected in 100 μl 1:1 serum free medium/Matrigel (BD Biosciences, San Jose, CA). Preparation of single cell suspensions was used for subsequent Xn propagation as described and for in-vitro experiments.

Primary tumor and propagated PDX (P2, P8, and P14) were authenticated by short tandem repeat profiling using several markers, including: D3S1358, vWa, D16S539, D2S1338, D8S1179, D21S11, D18S51, D19S433, TH01, and FGA.

### In vivo animal experiments

#### Estimation of the relative frequency of tumor propagating cells

To evaluate and compare the tumorigenic activity of Xn cells from early and late passages, serial dilutions (50−1 × 10^6^ cells) of cells suspended in 100 μl of PBS and 100 μl Matrigel were injected subcutaneously to the flank of NOD/SCID mice. Estimation of the relative frequency of cancer propagating cells was calculated online using the ELDA software online (http://bioinf.wehi.edu.au/software/elda/).

#### In vivo xenograft experiments using anti-NCAM1 antibody

1 × 10^6^ cells derived from late passage PPB PDX (P12) suspended in 100 μl 1:1 serum free medium/Matrigel were injected subcutaneously to the flank of NOD/SCID mice to generate tumors. Of the nine mice which developed a tumor, 4 were treated with 360 µg/Kg huN901-DM1^23^ while the other 5 were treated with saline as a control. Mice were treated intravenously twice weekly for a fortnight on days 0, 3, 7, and 11, and were observed for 17 days. Mice were monitored for tumor growth on days 0, 3, 7, 9, 11, 14. The investigator assessing Xn volume was blinded to the group allocation. On the 17^th^ day the mice were euthanized, tumors were harvested and measured. Tissue RNA was produced and used for qRT-PCR experiments.

### Fluorescence-activated cell sorting (FACS) analysis

FACS analysis of the primary PPB cells and subsequent fresh Xn derived cells was performed as previously described^14^. Surface markers antigens [CD24 (eBiosience, 120247-42), CD34 (Miltenyi, 3008100), CD56 (eBiosience, 1205942), CD90 (Beckman Coulter, IM3600U), H2K (eBiosience, 12-5958-82)] were labeled by incubation with fluorochrome conjugated antibody at a concentration of 5 µg antibody per 10^6^ cells for 30 min, in the dark, at 4 °C to prevent internalization of antibodies. In addition, we used 7-amino-actinomycin-D (7AAD; eBioscience, San Diego, CA) for viable cell gating. All washing steps were performed in FACS buffer. All Quantitative measurements were made in comparison to IgG isotype antibody.

### FACS sorting

FACS Aria was used to enrich for cells expressing surface markers. A 100-µm nozzle (BD Biosciences, San Jose, CA), sheath pressure of 20–25 pounds per square inch (PSI), and an acquisition rate of 1000–3000 events per second were used as conditions optimized for PPB cell sorting. Single viable cells were gated based on 7AAD, and then physically sorted according to NCAM1 expression into collection tubes for all subsequent experiments. Data was additionally analyzed and presented using FlowJo software.

### Microarray

The microarray data is deposited in publicly library (GEO); accession number GSE97236. All experiments were performed using Affymetrix HU GENE1.0st oligonucleotide arrays^49^. Total RNA from each sample was used to prepare biotinylated target cDNA, according to the manufacturer’s recommendations. The target cDNA generated from each sample was processed as per manufacturer’s recommendation using an Affymetrix Gene Chip Instrument System. Details of quality control measures can be found online. Significantly changed genes were filtered as changed by at least twofold (*p*-value: 0.05).

### Quantitative real-time reverse transcription PCR analysis–Gene expression analysis

Quantitative reverse transcription PCR (qRT-PCR) was carried out to determine fold changes in expression of a selection of genes. Total RNA from cells was isolated using an RNeasy Micro Kit (Qiagen GmbH, Hilden, Germany) according to the manufacturer's instructions. cDNA was synthesized using a High Capacity cDNA Reverse Transcription kit (Applied Biosystems, California USA) on total RNA. Real-time PCR was performed using an ABI7900HT sequence detection system (Perkin-Elmer/Applied Biosystems, California, USA) in the presence of TaqMan Gene Expression Master Mix (Applied Biosystems, California, USA). PCR amplification was performed using gene specific TaqMan Gene Expression Assay-Pre-Made kits (Applied Biosystems, California, USA). Each analysis reaction was performed in triplicate. HPRT1 or GAPDH were used as an endogenous control throughout the experimental analyses. PCR results were analyzed using SDS RQ Manager 1.2 software. Statistical analysis was performed using a non-paired 2-tails *T*-test. Statistical significance was considered at *P* < 0.05.

### H&E staining

H&E staining of 4 µm sections of paraffin-embedded tissues from primary PPB and propagated PDX were mounted on super frost/plus glass and incubated at 60 °C for 40 min. After deparaffinization, slides were incubated in Mayer's Hematoxylin solution (Sigma-Aldrich) and incubated with 1% HCl in 70% ethanol for 1 min. Slides were then incubated for 10 sec in Eosin (Sigma-Aldrich). Images were produced using Olympus BX51TF.

### Immunohistochemical staining of primary PPB and PPB Xn

Sections, 4 µm thick, were cut from primary PPB and PPB Xn for immunohistochemistry. Immunostainings were performed as previously described^50^. Briefly, the sections were processed to avoid oxidation of antigens. Before immunostaining, sections were treated with 10 mM citrate buffer, PH 6.0 for 10 min at 97 °C for antigen retrieval, followed by 3% H2O2 for 10 min. The slides were subsequently stained using the labeled strepavidin-biotin (LAB-SA) method using a Histostain plus kit (Zymed, San Francisco, CA, USA). The immunoreaction was visualized by an HRP-based chromogen/substrate system (liquid DAB substrate kit–Zymed, San Francisco, CA, USA). All antibody dilutions were carried out as recommended by the manufacturers of the staining antibodies. NCAM (Epitomics, cat #2433-1, 1:250), human HLA (Abcam, ab52922, 1:200), KI67 (Abcam, ab15580, 1:200), FGF5 (Abcam, ab89279, 1:50), FGF7 (Abcam, ab90259, 1 µg/ml), FGF10 (Abcam, ab80064, 1:20), Active caspase 3 antibody (Epitomics, cat #1476-1,1:10).

### PPB cell culture and in-vitro anti-NCAM1 experiment

Single cell suspension from PPB PDX tissues were grown in Bronchial Epithelial Growth medium (BEGM) (Lonza Walkersville, Inc. USA) and Iscove's Modification of Dulbecco's medium (IMDM) supplemented with 10% FBS and the following growth factors: EGF, FGF, and SCF (ratio 3:1 accordingly). Cells were treated with huN901-DM1^23^, a humanized anti-NCAM1 antibody-cytotoxic drug conjugate (ImmunoGen Inc., Waltham, Massachusetts) at a concentration of 0.18 µM. Following five days of treatment cells were harvested and RNA was derived for qRT-PCR experiments.

### Statistical analysis

Results are expressed as the mean ± S.E.M, unless otherwise indicated. Statistical differences in gene expression between PPB cell populations were evaluated using the Student's *T*-test. For animal studies, sample size was estimated to be at least four mice per group to ensure power with statistical confidence Statistical differences in the in-vivo experiments were calculated using Mann–Whitney *U* test. For all statistical analysis, the level of significance was set as *p* < 0.05 unless otherwise indicated.

## Supplementary information

Supplementary Information

Supplementary Information

## References

[1] Dishop MK, Kuruvilla S (2008). Primary and metastatic lung tumors in the pediatric population: a review and 25-year experience at a large children's hospital. Arch. Pathol. Lab. Med..

[2] Christosova IR (2015). Diagnosis and treatment of pleuropulmonary blastoma-single center experience. Pediatr. Pulmonol..

[3] Manivel JC (1988). Pleuropulmonary blastoma the so-called pulmonary blastoma of childhood. Cancer.

[4] Hill DA (2008). Type I pleuropulmonary blastoma: pathology and biology study of 51 cases from the international pleuropulmonary blastoma registry. Am. J. Surg. Pathol..

[5] Schultz Kris Ann, Yang Jiandong, Doros Leslie, Williams Gretchen M., Harris Anne, Stewart Douglas R., Messinger Yoav, Field Amanda, Dehner Louis P., Hill D. Ashley (2014). DICER1-Pleuropulmonary Blastoma Familial Tumor Predisposition Syndrome. Pathology Case Reviews.

[6] Doros, L. et al. in *DICER1-related disorders* (eds Adam M. P., Ardinger H. H., Pagon R. A., Wallace S. E., Bean L. J. H., Stephens K., et al.) (GeneReviews®, Seattle, 1993).

[7] Messinger YH (2015). Pleuropulmonary blastoma: a report on 350 central pathology-confirmed pleuropulmonary blastoma cases by the international pleuropulmonary blastoma registry. Cancer.

[8] Dehner LP (2015). Pleuropulmonary blastoma: evolution of an entity as an entry into a familial tumor predisposition syndrome. Pedia. Dev. Pathol..

[9] Pode-Shakked N, Dekel B (2011). Wilms tumor–a renal stem cell malignancy?. Pediatr. Nephrol..

[10] Shukrun R (2014). Wilms' tumor blastemal stem cells dedifferentiate to propagate the tumor bulk. Stem Cell Rep..

[11] Pode-Shakked N (2013). The isolation and characterization of renal cancer initiating cells from human Wilms' tumour xenografts unveils new therapeutic targets. EMBO Mol. Med..

[12] Pleniceanu O (2017). Peroxisome proliferator-activated receptor gamma (PPARgamma) is central to the initiation and propagation of human angiomyolipoma, suggesting its potential as a therapeutic target. EMBO Mol. Med..

[13] Golan H (2018). In vivo expansion of cancer stemness affords novel cancer stem cell targets: malignant rhabdoid tumor as an example. Stem Cell Rep..

[14] Dekel B (2006). Multiple imprinted and stemness genes provide a link between normal and tumor progenitor cells of the developing human kidney. Cancer Res..

[15] Metsuyanim S (2008). Accumulation of malignant renal stem cells is associated with epigenetic changes in normal renal progenitor genes. Stem Cells.

[16] Pode-Shakked N (2011). Resistance or sensitivity of Wilms' tumor to anti-FZD7 antibody highlights the Wnt pathway as a possible therapeutic target. Oncogene.

[17] Shukrun R, Pode Shakked N, Dekel B (2014). Targeted therapy aimed at cancer stem cells: Wilms' tumor as an example. Pediatr. Nephrol..

[18] Williams EJ, Furness J, Walsh FS, Doherty P (1994). Activation of the FGF receptor underlies neurite outgrowth stimulated by L1, N-CAM, and N-cadherin. Neuron.

[19] Cavallaro U, Niedermeyer J, Fuxa M, Christofori G (2001). N-CAM modulates tumour-cell adhesion to matrix by inducing FGF-receptor signalling. Nat. Cell Biol..

[20] Francavilla C (2007). Neural cell adhesion molecule regulates the cellular response to fibroblast growth factor. J. Cell Sci..

[21] Kos FJ, Chin CS (2002). Costimulation of T cell receptor-triggered IL-2 production by Jurkat T cells via fibroblast growth factor receptor 1 upon its engagement by CD56. Immunol. Cell Biol..

[22] Sanchez-Heras E, Howell FV, Williams G, Doherty P (2006). The fibroblast growth factor receptor acid box is essential for interactions with N-cadherin and all of the major isoforms of neural cell adhesion molecule. J. Biol. Chem..

[23] Whiteman KR (2014). Lorvotuzumab mertansine, a CD56-targeting antibody-drug conjugate with potent antitumor activity against small cell lung cancer in human xenograft models. MAbs.

[24] Tijink BM (2006). A phase I dose escalation study with anti-CD44v6 bivatuzumab mertansine in patients with incurable squamous cell carcinoma of the head and neck or esophagus. Clin. Cancer Res..

[25] Chari RV (1992). Immunoconjugates containing novel maytansinoids: promising anticancer drugs. Cancer Res..

[26] Erickson HK, Lambert JM (2012). ADME of antibody-maytansinoid conjugates. AAPS J..

[27] Ahmad I, Iwata T, Leung HY (2012). Mechanisms of FGFR-mediated carcinogenesis. Biochim. et. Biophys. Acta.

[28] Tenhagen M, van Diest PJ, Ivanova IA, van der Wall E, van der Groep P (2012). Fibroblast growth factor receptors in breast cancer: expression, downstream effects, and possible drug targets. Endocr. Relat. Cancer.

[29] Kochoyan A, Poulsen FM, Berezin V, Bock E, Kiselyov VV (2008). Structural basis for the activation of FGFR by NCAM. Protein science : a publication of the Protein. Society.

[30] Amoureux MC, Cunningham BA, Edelman GM, Crossin KL (2000). N-CAMbinding inhibits the proliferation of hippocampal progenitor cells and promotes their differentiation to a neuronal phenotype. J. Neurosci..

[31] Sugimoto KJ (2015). CD56-positive adult T-cell leukemia/lymphoma: a case report and a review of the literature. Med. Mol. Morphol..

[32] Takata K (2015). Primary cutaneous NK/T-cell lymphoma, nasal type and CD56-positive peripheral T-cell lymphoma: a cellular lineage and clinicopathologic study of 60 patients from Asia. Am. J. Surg. Pathol..

[33] Etzell JE, Keet C, McDonald W, Banerjee A (2006). Medulloblastoma simulating acute myeloid leukemia: case report with a review of "myeloid antigen" expression in nonhematopoietic tissues and tumors. J. Pediatr. Hematol./Oncol..

[34] Hinsby AM, Berezin V, Bock E (2004). Molecular mechanisms of NCAM function. Front. Biosci..

[35] Christensen C, Lauridsen JB, Berezin V, Bock E, Kiselyov VV (2006). The neural cell adhesion molecule binds to fibroblast growth factor receptor 2. FEBS Lett..

[36] Francavilla C (2009). The binding of NCAM to FGFR1 induces a specific cellular response mediated by receptor trafficking. J. Cell Biol..

[37] Kiselyov VV (2003). Structural basis for a direct interaction between FGFR1 and NCAM and evidence for a regulatory role of ATP. Structure.

[38] Davidson B (2015). The clinical role of epithelial-mesenchymal transition and stem cell markers in advanced-stage ovarian serous carcinoma effusions. Hum. Pathol..

[39] Flippot R, Kone M, Magne N, Vignot S (2015). [FGF/FGFR signalling: Implication in oncogenesis and perspectives]. Bull. du Cancer.

[40] He Q, Gong Y, Gower L, Yang X, Friesel RE (2016). Sef regulates epithelial-mesenchymal transition in breast cancer cells. J. Cell. Biochem..

[41] Mori S (2015). Enhanced expression of integrin alphavbeta3 induced by TGF-beta is required for the enhancing effect of Fibroblast Growth Factor 1 (FGF1) in TGF-beta-induced Epithelial-Mesenchymal Transition (EMT) in mammary epithelial cells. PLoS ONE.

[42] Takase N (2016). NCAM- and FGF-2-mediated FGFR1 signaling in the tumor microenvironment of esophageal cancer regulates the survival and migration of tumor-associated macrophages and cancer cells. Cancer Lett..

[43] Harris KS, Zhang Z, McManus MT, Harfe BD, Sun X (2006). Dicer function is essential for lung epithelium morphogenesis. Proc. Natl Acad. Sci. USA.

[44] Yin Y (2015). Fibroblast growth factor 9 regulation by MicroRNAs controls lung development and links DICER1 loss to the pathogenesis of pleuropulmonary blastoma. PLoS Genet..

[45] Zamai M (2019). Number and brightness analysis reveals that NCAM and FGF2 elicit different assembly and dynamics of FGFR1 in live cells. J. Cell Sci..

[46] Francavilla C (2019). Correction: The binding of NCAM to FGFR1 induces a specific cellular response mediated by receptor trafficking. J. Cell Biol..

[47] Zecchini S (2011). The adhesion molecule NCAM promotes ovarian cancer progression via FGFR signalling. EMBO Mol. Med..

[48] Turner N, Grose R (2010). Fibroblast growth factor signalling: from development to cancer. Nat. Rev. Cancer.

[49] Pode-Shakked N (2009). Developmental tumourigenesis: NCAM as a putative marker for the malignant renal stem/progenitor cell population. J. Cell. Mol. Med..

[50] Dekel B (2006). Isolation and characterization of nontubular sca-1+lin- multipotent stem/progenitor cells from adult mouse kidney. J. Am. Soc. Nephrol..

