# Supplementary material for: NCAM1/FGF module serves as a putative pleuropulmonary blastoma therapeutic target
Source: Oncogenesis. 2019 Sep 2;8(9):48. doi: 10.1038/s41389-019-0156-9 (PMC6718423; doi:10.1038/s41389-019-0156-9)
Supplement: Supplementary file 1 — Supplementary Information [file 41389_2019_156_MOESM1_ESM.doc]

**Supplementary Information**

**Figure S1**

**
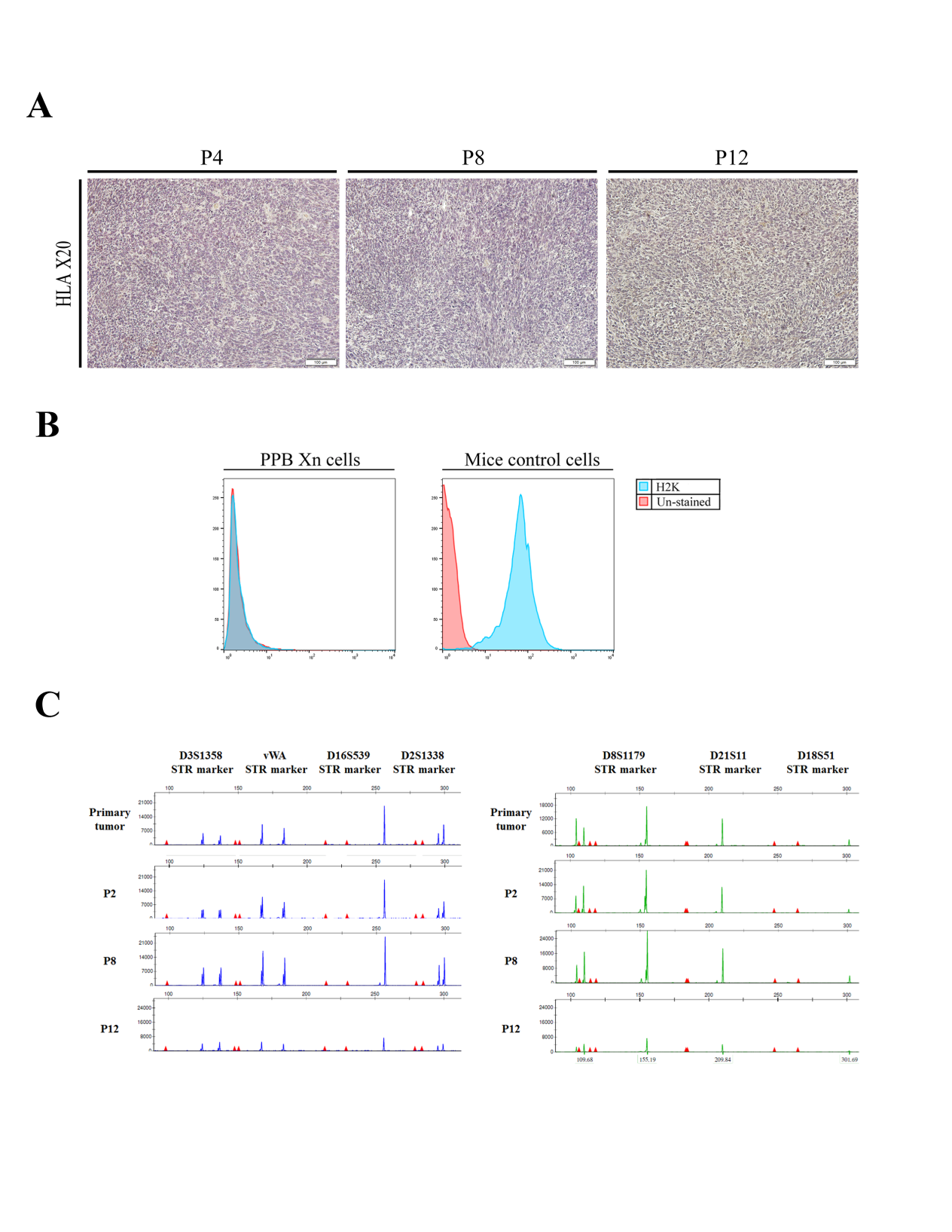
**

**(A)** PPB human HLA staining along passages demonstrating homogeneous layers of human PPB tumor cells. All cells from all passages were HLA positive, ruling out mice contaminant; Scale bar, 100μm. **(B)** Representative FACS analysis reveals that P14 PPB cells did not express H2K mice surface marker (left panel), while all control kidney mice cells are H2K+ (right panel). **(C)** Representative results of STR analysis from several tumor samples including primary tumor, P2, P8 and P12. STR, short tandem repeat.

**Figure S2**


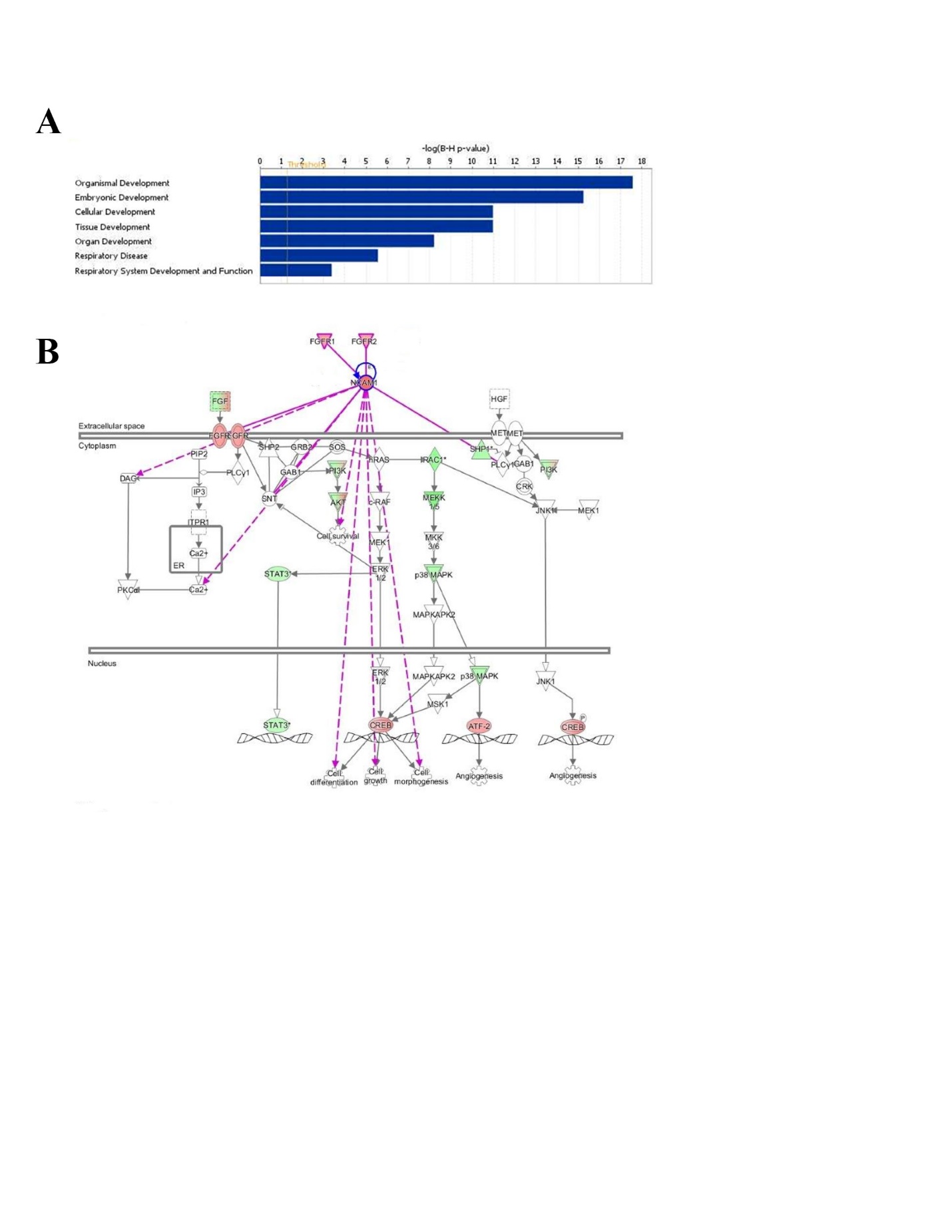


**(A)** Ingenuity functional analysis comparing P12 vs AL, demonstrating that among the most upregulated pathways are several developmental pathways including embryonic and respiratory development. **(B)** Ingenuity analysis scheme demonstrating the interaction between NCAM1, FGF receptors and several of their downstream targets;

**Figure S3**


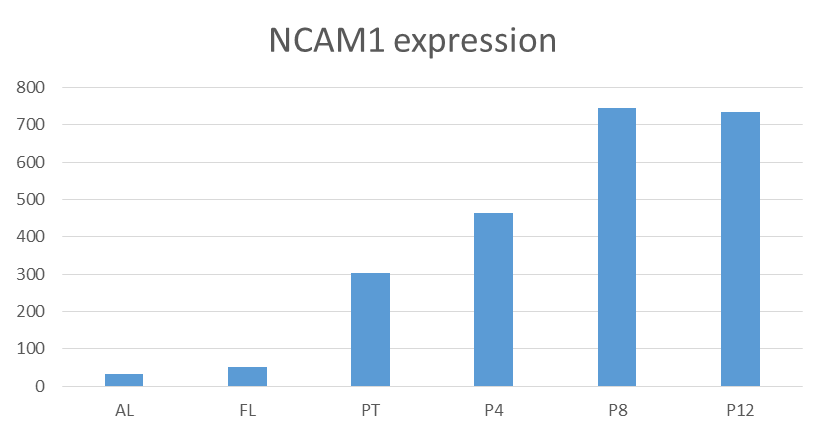


**(A)** Microarray gene expression analysis of NCAM1 expression comparing the different samples: 1. Adult lung (AL); 2. Fetal lung (FL); 3. Primary PPB (PT); 4. Early PPB PDX (Passage 4 - P4); 5. Intermediate PPB PDX (Passage 8 - P8); 6. Late PPB PDX (Passage 12 - P12); reveals an increase NCAM1 expression along the passages.

**Figure S4**

**
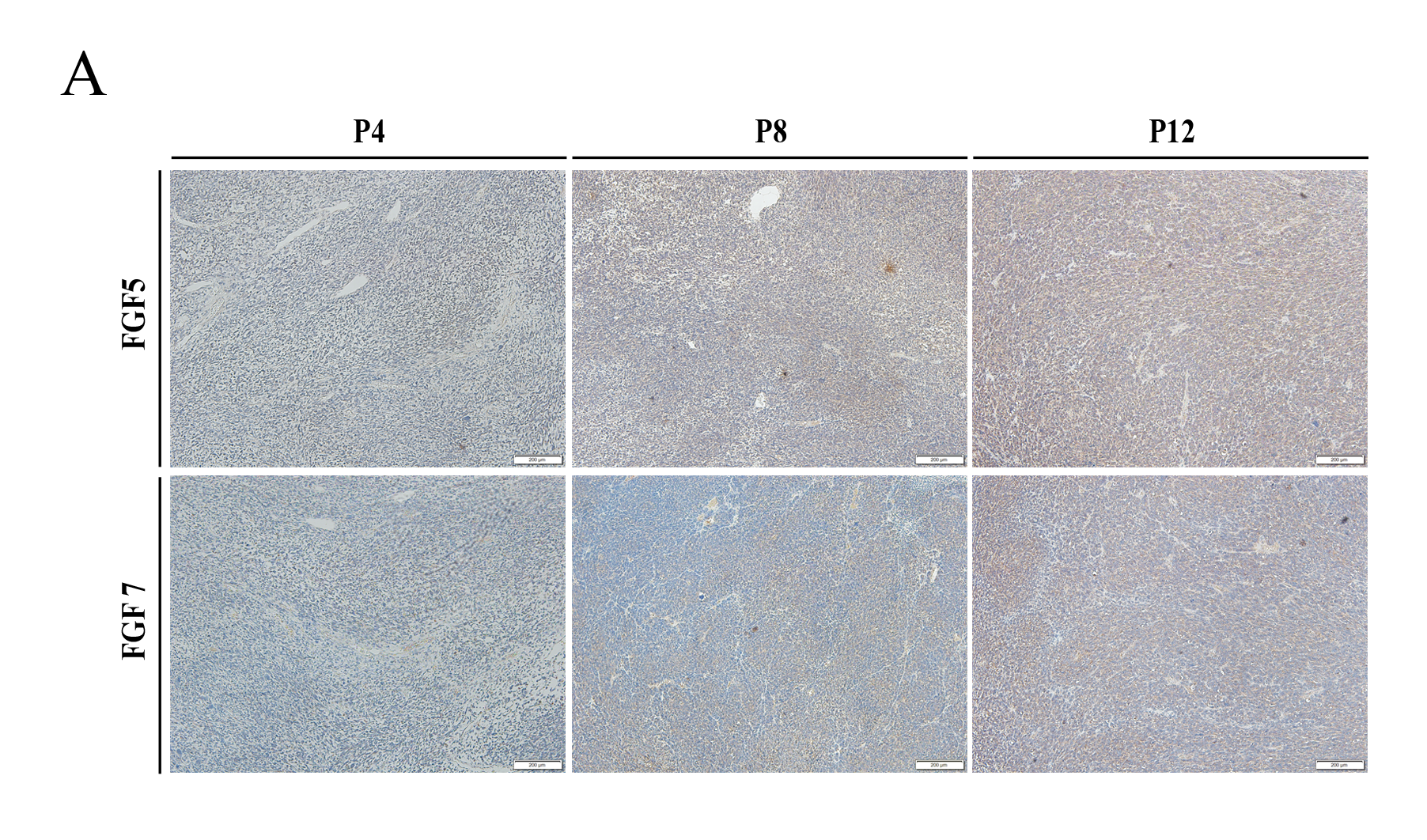
**

**(A)** IHC staining demonstrating an increased expression of FGF5 and FGF7 along the passages; Scale bar, 200μm.


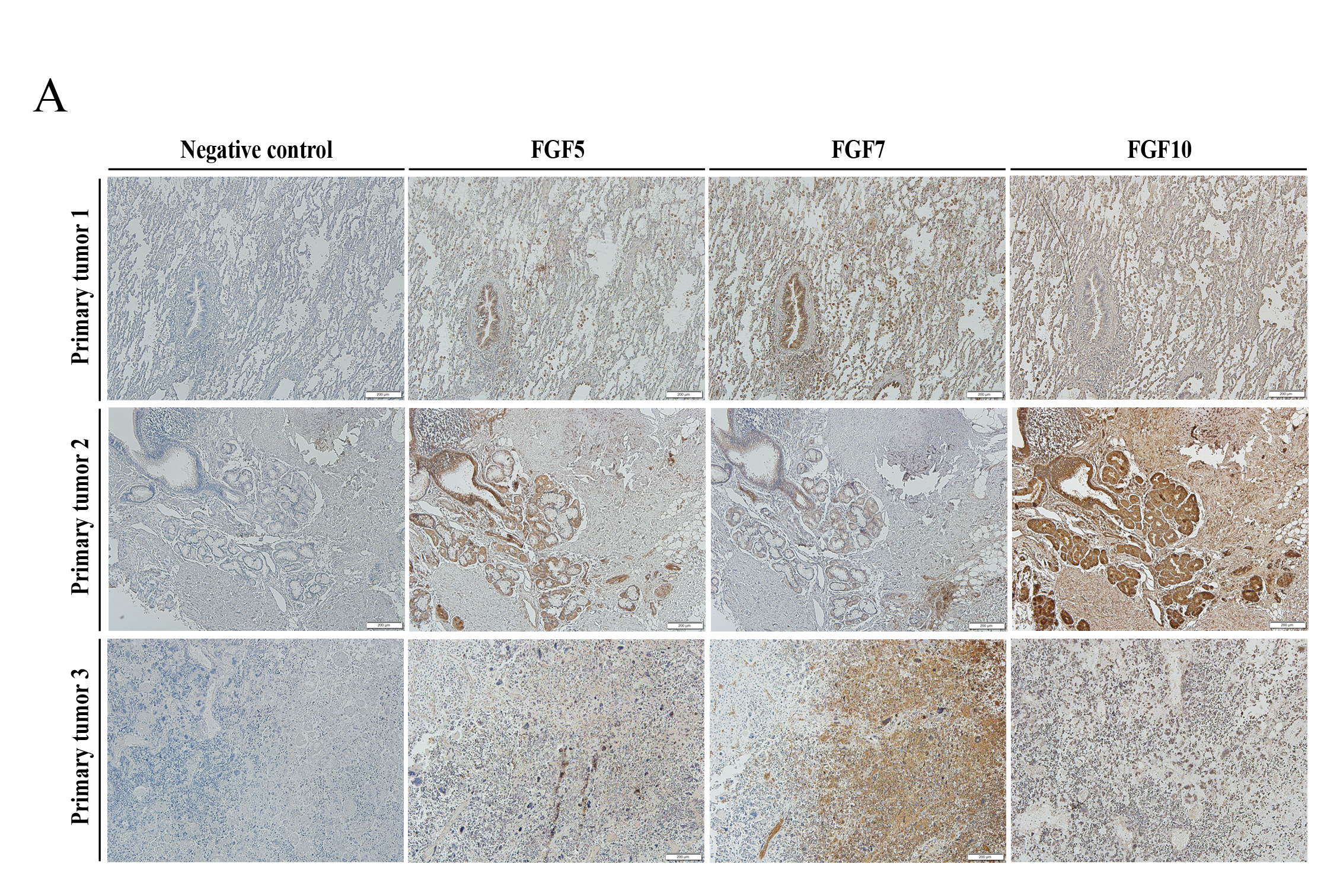
**Figure S5**

**(A)** IHC staining demonstrating FGF5, FGF7 and FGF10 expression in three different PPB primary tumors; Scale bar, 200μm.

**Figure S6**


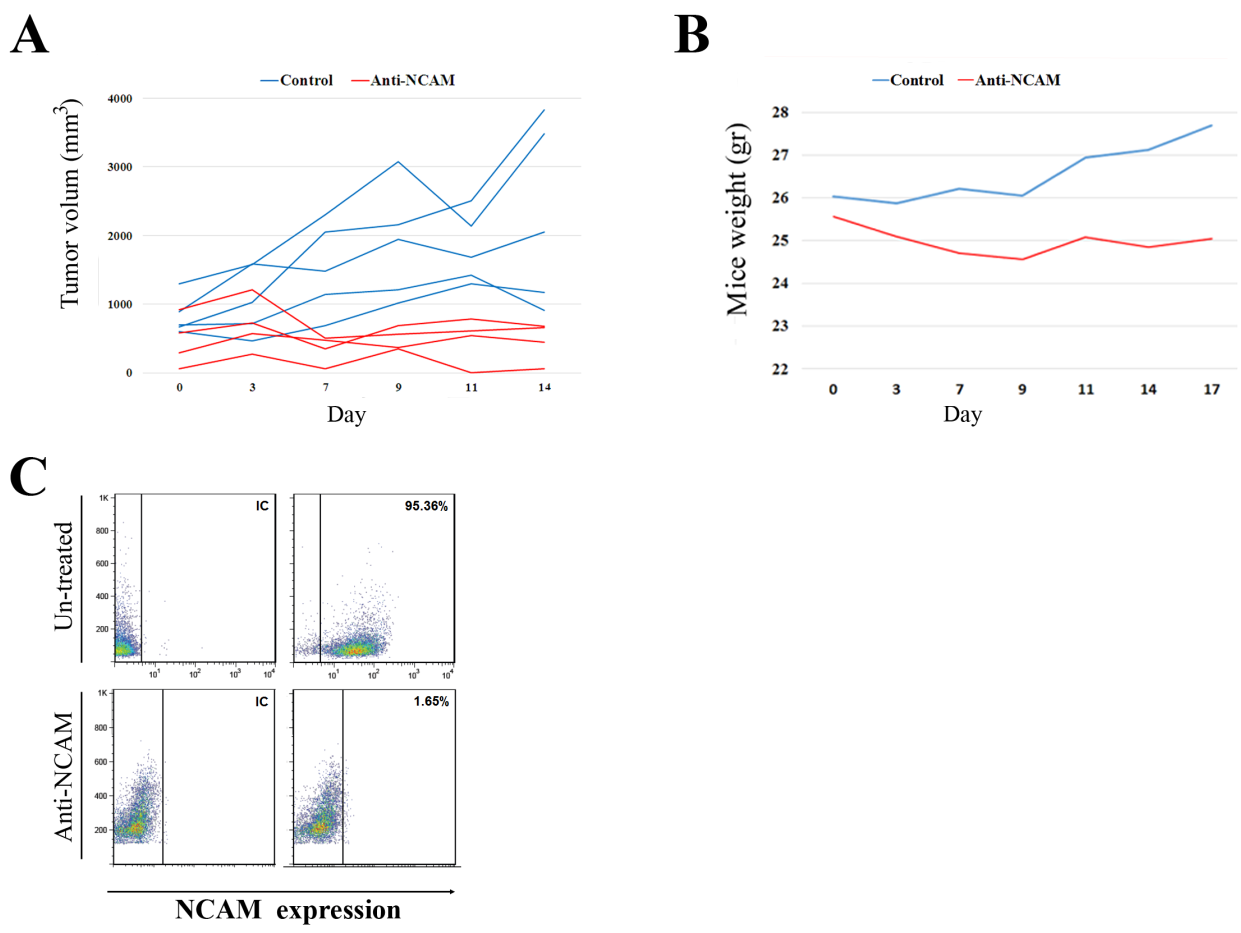
 **(A)** PPB Xn were formed and randomly divided into two groups, first group (N=4, red lines) was treated with lorvotuzumabmertansine (huN901-DM1) with a dosage of 360µg/Kg, while the second group (N=5, blue lines) was treated with saline as a control. Mice were treated intravenously twice weekly for a fortnight on days 0, 3, 7 and 11, and were observed for 17 days. In the Anti-NCAM1 treatment group, tumor volume was significantly lower in comparison to the control group following treatment. (B) A minor decrease in weight was observed in the huN901-DM1 treated group, while an eight percent weight gain was observed in the control group, most likely reflecting the significant increase in tumor volume. (**C)** FACS analysis demonstrating a significant downregulation in NCAM1 expression in the treated cells compared to the control group, 1.65% vs. 95.36% NCAM1 accordingly. IC, isotype control.

**Supplementary Tables**

**Table S1.** Late PPB Xn demonstrating a mesenchymal lung developmental signature.

| **Gene Symbol** | **Gene Title** | **P12 vs AL** | **PT vs AL** | **FL vs AL** |
| --- | --- | --- | --- | --- |
| GLI2 | GLI family zinc finger 2 | 127.00 | 51.68 | 12.40 |
| GLI3 | GLI family zinc finger 3 | 11.72 | 12.48 | 7.82 |
| PITX2 | paired-like homeodomain 2 | 209.07 | 247.83 | 1.95 |
| LEFTY1 | left-right determination factor 1 | 120.06 | 12.31 | 2.97 |
| SOX11 | SRY (sex determining region Y)-box 11 | 165.05 | 262.52 | 52.17 |
| SOX8 | SRY (sex determining region Y)-box 8 | 57.78 | 31.51 | 2.53 |
| MEST | mesoderm specific transcript homolog | 113.47 | 87.60 | 35.10 |
| PEG3 | paternally expressed 3 | 137.28 | 163.61 | 7.96 |
| PEG10 | paternally expressed 10 | 29.59 | 8.17 | 7.17 |
| NNAT | neuronatin | 112.41 | 35.75 | 1.76 |
| KCNQ1OT1 | KCNQ1 overlapping transcript 1 | 76.86 | 88.53 | 23.32 |
| DLK1 | delta-like 1 homolog (Drosophila) | 79.56 | 94.00 | 2.38 |
| IGF2 | insulin-like growth factor 2 | 83.54 | 47.37 | 4.45 |

Microarray gene expression analysis comparing several different samples: 1. primary PPB (PT); 2. Adult lung (AL); 3. Fetal lung (FL); 4. Passage 12 Xn (P12), demonstrating an early mesenchymal developmental signature including critical regulators of lung formation (e.g. *GLI2, GLI3, PITX2, LEFTY, SOX11, SOX8*) alongside paternally expressed genes (e.g. *PEG1/MEST, PEG3, PEG10, NNAT, KCNQ1OT1, DLK1* and *IGF2)* as previously shown for the WT blastemal.

**Table S2.** Activated signaling pathways and their downstream molecules involved in FGFR activation.

| **Pathway** | **Activated target genes** | | |
| --- | --- | --- | --- |
|  | **Gene symbol** | **PT vs AL** | **P12 vs AL** |
| **RAS-MAPK** | MYCN | 31.6 | 71.2 |
|  | KCNH2 | 14.3 | 36.2 |
|  | TNNI1 | 8.8 | 47.8 |
|  | POSTN | 5.8 | 57.9 |
|  | IRS1 | 4.1 | 9.0 |
|  | MKI67 | 3.4 | 5.0 |
|  | CTNNB1 | 3.2 | 4.9 |
|  | SNAI2 | 11.0 | 18.7 |
| **PIK3-AKT** | GLI2 | 5.5 | 12.8 |
|  | IGF2 | 47.4 | 83.5 |
|  | MYOD1 | 12.4 | 31.2 |
|  | CCNB1 | 10.4 | 20.7 |
|  | CCND2 | 9.9 | 12.4 |
|  | CCNE1 | 9.6 | 29.7 |
|  | BIRC5 | 9.3 | 17.3 |
|  | POSTN | 5.8 | 57.9 |
|  | COL1A1 | 7.9 | 8.6 |
|  | CDK4 | 7.6 | 11.8 |
| **STAT** | BEX1 | 21.5 | 48.2 |
|  | FST | 19.8 | 61.0 |
|  | SNAI2 | 11.0 | 18.7 |
|  | CCNB1 | 10.4 | 20.7 |
|  | FOXM1 | 10.4 | 18.7 |
|  | CCNE1 | 9.6 | 29.7 |
|  | BIRC5 | 9.3 | 17.3 |
|  | CDK1 | 8.6 | 14.6 |
|  | CDON | 8.6 | 28.0 |

Microarray gene expression analysis comparing several different samples: 1. primary PPB (PT); 2. Adult lung (AL); 3. Passage 12 (P12), demonstrating an upregulation of several FGFR’s signal pathways and their downstream targets.
